# Introduction to the Special Issue: Prevalence and Characteristics of Child Sexual Abuse Perpetration in Australia, the United States, and United Kingdom

**DOI:** 10.1177/08862605251403614

**Published:** 2026-03-03

**Authors:** Michael Salter, Delanie Woodlock, Tyson Whitten

**Affiliations:** 1 University of New South Wales, Sydney, Australia

**Keywords:** offenders/perpetrators, child maltreatment, prevention, child maltreatment, sexual abuse, child maltreatment, Internet and abuse, offenders/perpetrators, sexual assault

## Abstract

This special issue presents findings from the largest multi-country study of men’s self-reported sexual interest and behaviour towards children, addressing critical gaps in evidence on the prevalence, correlates, and prevention of child sexual exploitation and abuse perpetration. Drawing on three nationally representative male samples from Australia, the United Kingdom, and the United States, the seven papers provide the first cross-national estimates of undetected child sexual exploitation and abuse behaviour, identify demographic, psychological, developmental, and attitudinal factors associated with risk, and examine the role of online environments in facilitating harm. Collectively, the studies advance a public health approach to child sexual exploitation and abuse by delineating “upstream” strategies – such as early intervention, trauma prevention, social norms change, and digital regulation – that can reduce perpetration before it occurs. By integrating methodological, behavioural, and policy-oriented analyses, this special issue establishes a foundational evidence base to inform coordinated, cross-sectoral efforts to prevent child sexual exploitation and abuse and mitigate its profound community and intergenerational impacts.

## Introduction

This special issue presents comprehensive findings from the largest multinational study of men’s self-reported sexual interest and behaviour towards children. Collectively, these seven papers address critical gaps in knowledge about the prevalence and correlates of child sexual exploitation and abuse perpetration, with particular attention to three interrelated questions: (1) How many adult men in the general population sexually abuse children or are at risk of doing so? (2) What individual, social, and structural factors are associated with such behaviour? and (3) What policy and prevention opportunities exist to reduce perpetration at the community level? The evidence assembled here advances a growing international effort to conceptualise child sexual exploitation and abuse as a public health problem requiring coordinated “upstream” strategies that prevent abuse before it occurs.

Interest in public health approaches to child sexual exploitation and abuse reflects recognition of both the magnitude of the problem and the inadequacy of reactive ad hoc state responses. In Australia, for example, almost one in four people reports having been sexually abused in face-to-face settings (Mathews et al., 2023). Globally, 300 million children per annum experience some form of technology-facilitated child sexual exploitation and abuse (Fry et al., 2025). Survivors experience markedly elevated rates of mental health disorders (Scott et al., 2023), health risk behaviours such as substance misuse, self-harm, and suicidality (Lawrence et al., 2023), and substantially greater healthcare utilisation than the general population (Pacella et al., 2023). The impacts of child sexual exploitation and abuse are intergenerational and closely entwined with other forms of gender-based violence, including domestic and family violence (Salter et al., 2025). The provision of care, support, and safety is essential for survivors’ recovery; however, the burden of harm far exceeds the capacity of current health, social, and justice systems. Reducing this burden requires a complementary focus on preventing perpetration itself.

Despite investment in child sexual exploitation and abuse victimisation studies worldwide, comparable efforts to measure perpetration at the population level are rare. Most information about offending comes from forensic and clinical samples, which represent only a small fraction of those who harm children. Most offenders are never identified by authorities, and only a small proportion of detected offenders are charged or convicted. In Australia, just 16% of women and 1% of men who experienced child sexual abuse reported their first incident to police (Australian Bureau of Statistics, 2023). In one Australian jurisdiction, only 15% of child sexual exploitation and abuse cases known to police resulted in charges, and half of those led to conviction (Gilbert, 2025). Appeal rates for child sexual exploitation and abuse convictions are unusually high (Mills & Sharman, 2018). As a result, forensic and treatment-based samples represent a narrow and unrepresentative subset of offenders – typically those with poorer self-regulation, less capacity to evade detection, and fewer resources to defend themselves from criminal charges.

Measuring perpetration prevalence presents distinctive methodological challenges. Forensic samples and criminal justice statistics tend to under-count perpetration since offenders are very unlikely to self-report their full perpetration history to the authorities. While disclosure rates for child sexual abuse are low, victimisation studies provide an uneven indicator of offending prevalence in the community since one perpetrator may have multiple victims. Conventional public health methodologies such as household and telephone surveys are poorly suited to eliciting admissions of illegal or socially undesirable behaviour. Even anonymous telephone surveys have yielded negligible disclosure rates (Joyal & Carpentier, 2017). To obtain meaningful data, research designs must ensure full anonymity and create conditions conducive to perpetration disclosure, while simultaneously having a large enough reach to capture representative samples of the community.

To address this gap, the current study employed a large-scale, anonymous, online survey to obtain three independent samples of men aged 18 years or over representative of the Australian (*n* = 1,939), U.K. (*n* = 1,506), and U.S. (*n* = 1,473) adult male populations. The survey includes over 100 items measuring eight broad domains, including demographic factors, Internet use, physical and mental health, social adversity, and sexual feelings and behaviours towards children. Importantly, we found that a non-trivial proportion of men reported sexual behaviours, feelings, and interests towards children: 9.2% reported interest in technology-facilitated child sexual exploitation and abuse and 6.0% had engaged in it; 7.3% would have sexual contact with a child while 4.5% actually had; 10.1% were sexually attracted to children under 16 years and 6.0% to children under 11 years; and 5.7% were concerned about their sexual feelings towards children and wanted help. This study provides the first cross-national dataset on perpetration prevalence, correlates, and attitudes, allowing systematic comparison of how social, political, and institutional contexts shape risk. Unlike forensic and treatment-oriented data that dominates offender research, this study was designed to inform public policy by identifying the social, structural, and commercial correlates of child sexual exploitation and abuse perpetration in community settings.

Conducting a cross-jurisdictional study of this nature introduced conceptual and legal complexities. The age of consent varies between and within countries, and inconsistencies persist between online and offline offences. To ensure comparability, the study adopted the international legal definition of a child – under 18 years – as a uniform threshold across all offence types. This approach aligns with global child protection norms, providing definitional consistency while acknowledging some loss of jurisdictional specificity. Future iterations may incorporate more granular items to capture contextual variation, especially as emerging privacy-preserving technologies (including AI-enabled research interfaces) enhance opportunities to study sensitive and illegal behaviour. The study also highlights unresolved conceptual debates within the field, particularly regarding the measurement and terminology of sexual interest in children, its dimensionality, and its underlying mechanisms. There is a clear need for ongoing, large-scale community-based perpetration research to refine measures of sexual interest and behaviour towards children, while acknowledging the ambiguities and grey areas inherent in legal definitions.

The seven papers in this special issue represent a series of exploratory analyses that collectively trace the methodological, developmental, psychological, and policy dimensions of child sexual exploitation and abuse perpetration. The opening paper details the study’s sampling strategy, survey design, and analytic procedures across three nationally representative male cohorts. Building on this foundation, the second paper, “Exploring Men’s Self-Reported Sexual Feelings, Interests, and Behaviours Towards Children,” presents the first population-level estimates of men’s sexual interest in and offending against children across the three countries. It delineates the extent of undetected behaviour, co-occurrence patterns, and demographic correlates, revealing distinct clusters of online behaviours, contact offending, and sexual interest. These findings support a targeted and coordinated approach to prevention that distinguishes sexual interest from sexual behaviour towards children and seeks to target men at risk of abusing children as early as possible. This paper also highlights a consistent theme throughout the study, namely that men who sexually abuse children are disproportionately likely to report sociodemographic indicators linked to social and economic capital and access to children.

The third paper, “Differentiating Men With Sexual Interest in Children and Those Involved in Sexual Behaviour With Children,” is an empirical exercise in using penalised regression with cross-validation to identify the factors associated with distinct categories of child sexual exploitation and abuse behaviour and interest: (a) those with neither interest nor behaviour, (b) interest only, (c) behaviour only, and (d) both interest and behaviour. Multivariable analyses identified attitudinal, psychological, and behavioural factors that differentiate these groups, particularly highlighting the distinct profile of men with both sexual interest and offending behaviour. This step refines the typology of potential offenders and clarifies that sexual interest alone is neither a necessary nor sufficient condition for offending, underscoring the importance of other mediators.

The fourth paper, “Identifying the Demographic and Internet Use Characteristics of Technology-Facilitated Child Sex Offenders Operating in the Australian, U.S. and U.K. General Population,” is a descriptive analysis of the prevalence and correlates of technology-facilitated child sexual exploitation and abuse, situating these exploratory findings within regulatory and policy frameworks that shape online behaviour. By identifying demographic and online behavioural patterns associated with technology-facilitated offending, this paper bridges offline and online offending domains and highlights the role of online services and platforms in amplifying risks and opportunities for harm. The paper points to the significantly higher rates of online offending in the US compared to the other two jurisdictions and considers the role of internet regulation as a primary prevention strategy for technology-facilitated child sexual exploitation and abuse.

The next paper, “Examining the Cross-Country Differences in the Adverse Childhood Experiences Associated With Men Who Have Sexual Feelings and Offend Against Children Online,” explores the association between men’s adverse childhood experiences (ACE) and likelihood of engagement or interest in technology-facilitated child sexual exploitation and abuse. The role of childhood trauma and adversity in the perpetration of child sexual abuse is a long-standing debate in the field, and this paper sought to examine this association with a focus on technology-facilitated abuse. The paper identifies distinct patterns of individual and cumulative ACEs associated with both interest and behaviour, with the strongest associations for childhood sexual abuse, neglect, and emotional abuse. The cross-country comparisons suggest contextual variation in how early-life adversity translates into adult offending risk, providing evidence for upstream prevention through child protection, trauma-informed, and early-intervention strategies.

The sixth paper, “Examining Attitudes Conducive to Child Sex Offending: Evidence From a Representative Multi-country Study”, turns to cognitive and normative dimensions, analysing latent classes of men’s attitudes towards child sexual exploitation. Primary prevention strategies to reduce violence and abuse have often focused on changing norms and attitudes; however, there has been limited attitudinal research conducted to date that identifies attitudes associated with child sex offending. This paper shows that attitudinal configurations differ markedly between men who have offended, those with sexual interest only, and those without either. This paper identifies the attitudinal scaffolding that normalises or excuses abuse, offering crucial insights for social norms interventions aimed at disrupting cognitive pathways to offending.

The final paper, “A Descriptive Study of the Characteristics Differentiating Men Who Do and Do Not Want Help for Their Sexual Feelings Towards Children,” translates the preceding findings into prevention practice. It examines the characteristics of men who want help for their sexual interest in children versus those who do not, noting that men with a sexual interest in children who want help are more likely to have already committed an offence compared to those who do not want help. However, the group who wanted help were also more likely to be married or living with a partner, with closer social bonds, suggesting that help-seeking may also be motivated by concern about relational and reputational harm should their offending be exposed. The paper reflects on the implications of these findings for increasing men’s motivations for seeking help prior to offending onset.

In sum, the papers in this special issue collectively advance a transformative research agenda for understanding and preventing child sexual exploitation and abuse. By integrating prevalence data, attitudinal and behavioural typologies, developmental antecedents, and the dynamics of online and offline offending, the collection reframes child sexual exploitation and abuse as a multifactorial public health problem rather than solely a matter of individual pathology. The findings underscore the importance of upstream prevention – addressing early adversity, reshaping harmful social norms, strengthening digital regulation, and expanding access to non-judgemental support for those at risk of offending. Together, these studies provide an unprecedented empirical foundation for coordinated, cross-sectoral strategies to reduce the perpetration of child sexual exploitation abuse and its profound harms across generations.
